# SARS-CoV-2 transmission, the ambiguous role of children and considerations for the reopening of schools in the fall

**DOI:** 10.2217/fmb-2020-0195

**Published:** 2020-09-03

**Authors:** Cleo Anastassopoulou, Nicholas Spanakis, Athanasios Tsakris

**Affiliations:** ^1^Laboratory of Microbiology, Medical School, National & Kapodistrian University of Athens, Athens, Greece

**Keywords:** children, COVID-19, novel coronavirus, pediatric cases, reopening, SARS-CoV-2, schools, transmission

## The unknown correlates of severe acute respiratory syndrome coronavirus 2 transmission

One of the key characteristics of severe acute respiratory syndrome coronavirus 2 (SARS-CoV-2) is high transmissibility which, fueled by globalization, led to its rapid spread among the worldwide human population since its emergence in Wuhan, Hubei Province, China in late December 2019 [1]. Within a period of four months, the resulting coronavirus disease of 2019 (COVID-19) was declared a pandemic by the WHO and war-state images of healthcare workers in full personal protective equipment gear and impromptu hospitals, reminiscent of historic epidemics like the influenza of 1918, revived in response to this unprecedented for our times public health crisis [2]. Ironically, in the era of the Fourth Industrial Revolution and artificial intelligence, centuries-old interventions, such as face coverings, social distancing and isolation (quarantine) of infected cases, as well as shutdown of cities, regions or whole countries, were implemented to contain the spread of the seventh coronavirus known to infect humans, violently disrupting societal and economic activities across the globe. And as countries, regions or states were taking the first steps toward reopening after strict lockdowns, the resurgence of cases at ever shifting epicenters across different continents prompted a top official from the WHO to warn that the world has entered a “new and dangerous phase” of the COVID-19 pandemic on 19 June 2020 [3].

Similarly to the other two epidemic coronaviruses that can be fatal for humans, the first severe acute respiratory syndrome coronavirus (SARS-CoV-1) of 2002–2003 and the Middle East respiratory syndrome coronavirus of 2012, the origin of SARS-CoV-2 is thought to be zoonotic, possibly starting from bats [4,5]. Coronaviruses are characterized by frequent cross-species transmission events, which very likely are accompanied by changes in virus tropism, whether they be from animal-to-human (zoonoses), animal-to-animal or human-to-animal (reverse zoonoses) [6]. At present, the risk of animals transmitting the novel coronavirus to people is considered to be low. Under experimental conditions, while testing for suitable animal models of COVID-19, the virus has been shown to infect rhesus and cynomolgus macaques, ferrets, cats and golden Syrian hamsters, with some of these animal species (rhesus macaques, juvenile cats and hamsters) displaying mild-to-moderate clinical disease [7–13]. In addition to these experimental infections, there have been reports of occasional spillover from infected humans to domestic (cats and dogs) and captive animals (tigers and farmed mink), but there is no evidence that animals play a significant role in spreading the virus [14–16].

Up until very recently, SARS-CoV-2 was thought to be spreading from person-to-person principally through respiratory droplets generated during coughing and sneezing by infected individuals and deposited on persons or objects for continuation of the transmission chain by direct or indirect contact, respectively. Aerosol and fomite transmission of both SARS coronaviruses leading to super-spreading events is plausible [17]. However, accumulating evidence shows that, in contrast to SARS-CoV-1, SARS-CoV-2 is transmitted before the onset of symptoms by symptomatic persons and even by asymptomatic individuals [18,19]. Droplets of saliva and other secretions are expelled from the respiratory tract (the mouth and nose) not only during such obviously forceful expiratory activities as sneezing and coughing, but also during breathing, particularly during intense physical exercise, talking, laughing and singing. We cannot help but wonder whether the extrovert nature of people from the south of Europe, who typically speak loudly and have increased and often demonstratively affectionate social contacts not only with significant others, but also with friends and acquaintances and, notably, with extended family members, including elderly grandparents, has contributed to the devastating trajectories of the epidemic in countries like Italy and Spain.

Respiratory exhalations do not solely consist of mucosalivary droplets that follow short-range semi-ballistic emission trajectories allowing for a dichotomy between large and small droplets, as the still adopted by the WHO and other agencies transmission model originally proposed by William F Wells for tuberculosis in the 1930s had suggested. Instead, according to the new model for respiratory emissions, exhalations consist of clusters of varying in size droplets, trapped in a multiphase turbulent gas (a puff) cloud of a locally moist and warm atmosphere that prevents evaporation, thereby considerably extending the time and distances virus particles can travel [20]. The implication of the current model is that a multiplicity of biophysical parameters determines the rates at which droplets of all sizes settle out or evaporate, depending not only on their size, but also on the degree of turbulence and speed of the gas cloud, coupled with the properties of the ambient environment (temperature, humidity and airflow), further perplexing the matter. Airborne transmission of aerolized virus particles, which has just been identified as an additional, if not the dominant, spreading route of COVID-19, appears to have contributed to the successful propagation of the novel coronavirus [21]. This latest finding prompted 239 scientists from 32 countries to urge the WHO and other bodies to address this potential in conjunction with contact transmission and mandate face covering in public to mitigate the airborne microdroplet transmission, in an open letter published on 6 July [22]. Hence, the correlates of the sustained human-to-human transmission of SARS-CoV-2 have not been clearly defined yet.

## Controversies in pathogenesis & the ambiguous role of children in transmission

School closures that are commonly suggested for mitigating epidemics of respiratory infection were among the first social distancing measures implemented during this pandemic. Decreases in COVID-19 cases were indeed noted after school and daycare closures in many communities, even though modeling studies predicted that school closures alone would prevent only 2–4% of deaths, much less than other social distancing interventions, according to a review from the UK led by University College London [23].

From the early phases of the outbreak, it became apparent that adult pathogenesis varies with age and gender since older men with comorbidities (e.g., diabetes or cardiovascular disease) were at higher risk for severe illness from COVID-19 [24–26]. At the other end of the spectrum, children generally experience less severe disease and have better prognosis after infection with SARS-CoV-2 [27], although a multisystem inflammatory syndrome (MIS-C) resembling Kawasaki Disease and toxic shock syndrome-like conditions has been observed in pediatric patients [28]. In addition, infants and preschool-aged children may be prone to more significant illness than older children [29,30]. Several explanations have been suggested for the typically low morbidity and mortality in children, including fewer underlying medical conditions and healthier respiratory tracts because of less exposure to cigarette smoke and air pollution and stronger innate immune responses [31]. An alternative explanation for the generally benign disease course in pediatric cases pertains to the less mature, and thus of decreased functionality (e.g., binding potential)-ACE2 protein receptors for SARS-CoV-2 entry into cells. In agreement with this reasoning, data suggest that SARS-CoV-2 infections in children involve the upper rather than the lower respiratory tract, the typical site of severe COVID-19 disease where ACE2 receptors are more abundant [29].

The role of children in the transmission of the novel coronavirus is unclear [32]. By fitting an age-structured mathematical model to epidemic data from China, Italy, Japan, Singapore, Canada and South Korea, Davies *et al*. estimated that susceptibility to infection in individuals under 20 years of age was approximately half that of adults aged over 20 years and that interventions aimed at children might have a relatively small impact on reducing SARS-CoV-2 transmission, particularly in settings where the transmissibility of subclinical infections is low [33]. Studies from Israel and Greece that examined the dynamics of COVID-19 transmission within families showed significantly lower infection rates in children compared with adults residing in the same household [34,35]. In a study of young children from northern France [36], SARS-CoV-2 infection was found to be largely mild or asymptomatic and there was no evidence of onwards transmission from children in the school setting in line with previous studies from Australia, Ireland and France [37–39]. Primary or elementary school children aged 6–11 years were less likely to transmit the virus compared with high school aged children who could transmit the virus as efficiently as adults. A large, carefully conducted study from South Korea corroborated these results [40]. The reason why children younger than 10 years might transmit to others much less often than adults could be because of the air volume, and therefore of the virus-laden air, normally exhaled by children that is smaller or closer to the ground, making it less likely that adults would breathe it in. Older children in middle and high school, who can be as tall as adults and yet may have some of the same unhygienic habits as young children, were found to be even more likely to infect others compared with adults by this South Korean study [40].

Analysis of 3303 COVID-19 pediatric patient sputum samples by Christian Drosten’s group in Germany using two different PCR systems to assess viral load, determined that children under the age of 19 produce virtually the same average levels of viral RNA as adults [41]. Another study from Chicago showed that children younger than 5 years had tenfold to 100-fold greater amount of SARS-CoV-2 viral RNA, which correlates with infectious virus [42], in their upper respiratory tracts compared with older children aged 5–17 years or adults aged 18–65 years [43]. Sampling bias due to the increased possibility of asymptomatic infection in children that reduces the chances of being tested early in infection when virus levels in the nasopharynx are high, could explain the disparity in results from different studies. Since viral RNA levels in the feces remain high for more than three weeks after the onset of symptoms in contrast to viral RNA in the nose and saliva that decline drastically within 1–2 weeks, analyzing stool samples for transmissible virus from mildly symptomatic and asymptomatic children could provide the experimental data to validate this argument [44]. Viral replication in the GI tract and prolonged fecal shedding by infected children, particularly for infants and younger children who are not toilet-trained, may have substantial implications for community spread in day-care centers, schools and homes [29,32].

Further studies are needed to clarify the role of children in the transmission of the novel coronavirus. In this respect, a large prospective NIH-funded study of 6000 people from 2000 US families in 11 cities, called human epidemiology and response to SARS-CoV-2, will help determine the incidence of novel coronavirus infection in children in the USA and whether rates differ between children who have asthma or other allergic conditions and children who do not [45].

## Considerations for the reopening of schools in the fall

The new US Centers for Disease Control and Prevention (CDC) guidelines on education and child care support the opening of schools in the fall. The main arguments in favor of the return to in-person schooling come from early reports that suggest that children are less likely to be infected than adults (at least based on nasopharyngeal viral RNA detection as discussed in the previous subsection), and when they do contract COVID-19, they generally have less serious illness. As of 21 July 2020, children and adolescents under 18 years old accounted for under 7% of COVID-19 cases and less than 0.1% of COVID-19-related deaths in the USA.

Schools, an important part of the infrastructure of communities, are indeed critical for the socialization and education of children, especially for students who rely on special education services (e.g., speech and occupational therapy). However, the evidence about whether returning to school results in increased transmission or outbreaks is inconclusive. Transmission in schools appears to mirror rates in the community and, therefore, school reopenings seem to be safe when community SARS-CoV-2 transmission rates are low, as shown by international studies that have assessed how readily COVID-19 spreads in schools [46].

School children are nonetheless anticipated to contribute to the community transmission of SARS-CoV-2 through their large numbers of daily social contacts, some of which are intergenerational, with older age groups where the risk for more severe illness is increased. Hence, the reopening of schools should be considered carefully, with continuous monitoring of possible resurgence in infections through frequent testing and isolation of infected – and perhaps more importantly of infectious – students, educators and other staff, for their safety and for the safety and wellbeing of their families. The current Centers for Disease Control and Prevention guidelines do not recommend universal screenings by schools; instead, they strongly encourage parents or caregivers to monitor their children every day for ‘signs of infectious illness’, most likely referring to symptoms that, if present at all, are perceived differently by infected individuals, and to keep sick students at home.

When schools reopen, additional strategies to limit transmission should be implemented as at other essential workplaces, including hand hygiene, physical distancing considering the recent advances in the biophysics of host-to-host respiratory disease transmission, respiratory etiquette and masks for older children. Such mitigation measures would also help contain other respiratory infections in the community. Alternative plans for virtual learning should be in place in the event of a school closure if community transmission levels cannot be controlled. Finally, an alternative consideration that could provide both direct and indirect benefit given that children may contribute to adult infections and develop life-threatening disease themselves, is that vaccine development efforts for COVID-19 should perhaps also consider the pediatric population.

Until we have safe and efficient prophylactic vaccines or therapeutics, or until SARS-CoV-2 becomes endemic, we have to continue to expect the unexpected from this novel coronavirus, the even brief encounter of which with our species, has changed the world forever.
